# Association Between Body Composition, Muscle-to-Weight Ratio, and Functional Disability in Patients with Chronic Non-Specific Low Back Pain: A Cross-Sectional Study

**DOI:** 10.3390/medicina62081474

**Published:** 2026-07-29

**Authors:** Sebastian Tirla, Anamaria Gherle, Laura Ioana Bondar, Brigitte Osser, Victor Niculescu, Diana Carina Iovanovici, Cristian Marge, Felicia Liana Andronie-Cioara, Carmen Delia Nistor-Cseppento

**Affiliations:** 1 Doctoral School of Biomedical Sciences, Faculty of Medicine and Pharmacy, University of Oradea, 410073 Oradea, Romania; tirla.sebastian@student.uoradea.ro (S.T.); gherle.anamaria@student.uoradea.ro (A.G.); dcseppento@uoradea.ro (C.D.N.-C.); 2 Department of Psycho Neuroscience and Recovery, Faculty of Medicine and Pharmacy, University of Oradea, 410073 Oradea, Romania; marge.cristian@uoradea.ro (C.M.); fcioara@uoradea.ro (F.L.A.-C.); 3 Department of Biology and Life Sciences, Faculty of Medicine, “Vasile Goldiș,” Western University of Arad, 310048 Arad, Romania; 4 Faculty of Physical Education and Sport, “Aurel Vlaicu” University of Arad, 310130 Arad, Romania; 5 Department of Fundamental, Preventive, and Clinical Disciplines, Faculty of Medicine, Transilvania University of Brașov, 500036 Brașov, Romania; victorniculescu91@gmail.com; 6 Institute of Cardiovascular Diseases Timișoara, 300310 Timișoara, Romania; diana_iovanovici@yahoo.com

**Keywords:** body composition, disability evaluation, low back pain, pain measurement, rehabilitation, Roland–Morris Disability Questionnaire, muscle composition, muscle-to-weight ratio

## Abstract

*Background/Objectives:* Low back pain (LBP) is a leading cause of disability worldwide and is influenced by multiple biological and functional factors. While pain intensity is a well-established determinant of disability, the contribution of body composition and relative skeletal muscle mass remains insufficiently understood. This study aimed to investigate the associations between body composition parameters, Muscle-to-Weight Ratio (MWR), pain intensity, and functional disability in patients with LBP and to explore sex-related differences in these variables. *Methods:* A cross-sectional observational study was conducted in 98 adults with chronic non-specific LBP recruited from a rehabilitation department in Romania. Anthropometric and body composition measurements were obtained using bioelectrical impedance analysis (BIA). Pain intensity was assessed using the Visual Analogue Scale (VAS), and functional disability was evaluated using the Roland–Morris Disability Questionnaire (RMDQ). The MWR was calculated as skeletal muscle mass divided by body weight and expressed as a percentage. Sex comparisons, correlation analyses, and multiple linear regression analyses were performed. *Results:* The mean age of participants was 61.9 ± 10.0 years, and 53.1% were female. Female participants reported significantly higher pain intensity than males (60.5 ± 31.0 vs. 44.2 ± 35.5; *p* = 0.021) and demonstrated a different distribution of disability severity categories compared with males (Fisher–Freeman–Halton exact *p* = 0.021). Pain intensity showed the strongest positive correlation with disability (r = 0.56, *p* < 0.001), whereas MWR was negatively correlated with disability (r = −0.34, *p* = 0.001). In multivariable regression analysis, pain intensity (β = 0.49, *p* < 0.001), age (β = 0.22, *p* = 0.013), and MWR (β = −0.27, *p* = 0.018) remained significantly associated with disability after adjustment for sex and body fat percentage. The model explained 43.2% of the variance in RMDQ scores (R^2^ = 0.432). *Conclusions:* Pain intensity, age, and MWR were significantly associated with functional disability in patients with chronic non-specific LBP. Lower MWR values were associated with greater disability after adjustment for age, sex, body fat percentage, and pain intensity. These findings suggest that assessment of body composition and MWR may provide additional information when evaluating patients with chronic non-specific low back pain. However, the cross-sectional design precludes conclusions regarding causality.

## 1. Introduction

Low back pain (LBP) is one of the most prevalent musculoskeletal disorders worldwide and remains a leading cause of disability across all age groups. It is estimated that up to 80% of individuals experience at least one episode of LBP during their lifetime, resulting in substantial personal, social, and economic burdens [1,2,3,4]. Beyond pain, LBP is frequently associated with reduced physical function, impaired quality of life, work absenteeism, and increased healthcare utilization [5,6,7,8]. Given its multifactorial etiology, identifying factors associated with disability among patients with LBP is essential for improving clinical management and rehabilitation outcomes.

LBP is broadly classified as specific or non-specific according to its underlying etiology. Approximately 90–95% of cases are classified as non-specific LBP, in which no specific pathoanatomical cause can be identified. Furthermore, symptoms are commonly categorized according to duration as acute (<6 weeks), subacute (6–12 weeks), or chronic (>12 weeks). Chronic non-specific LBP represents the most prevalent clinical subtype and is associated with persistent pain, functional disability, and substantial socioeconomic burden [9,10,11].

The development and persistence of disability in patients with LBP are influenced by a complex interaction of biological, biomechanical, and psychosocial factors. Pain intensity has consistently been identified as one of the strongest determinants of functional limitation [12,13,14,15,16]. However, increasing evidence suggests that body composition characteristics may also be associated with the severity of symptoms and disability. Obesity has been associated with chronic low-grade inflammation, increased mechanical loading of the spine, reduced mobility, and poorer clinical outcomes among individuals with LBP [17,18,19]. Nevertheless, conventional anthropometric indicators such as body mass index (BMI) may not adequately reflect alterations in body composition that influence physical function.

Recent attention has focused on skeletal muscle health and body composition in patients with chronic non-specific LBP. Reduced skeletal muscle mass and unfavorable body composition have been associated with impaired physical function and disability. Sarcopenia, which represents the severe clinical manifestation of impaired muscle health, has also been associated with these outcomes [20,21,22,23]. Emerging evidence indicates that reduced muscle quantity and quality are associated with greater pain intensity, poorer physical performance, and increased functional disability in patients with chronic non-specific LBP [24,25,26]. In addition, recent observational studies and systematic reviews have shown that unfavorable body composition characteristics, including reduced skeletal muscle mass and increased adiposity, may contribute to pain persistence and impaired physical function in this population [27,28]. Consequently, muscle-related indices may provide clinically relevant information beyond traditional anthropometric measures when evaluating patients with chronic non-specific LBP.

Among the available body composition indicators, the Muscle-to-Weight Ratio (MWR) has been proposed as a practical measure of muscle status and may better capture the balance between muscle and adipose tissue than isolated measurements of muscle mass alone. Although previous studies have demonstrated associations between sarcopenia, obesity, or individual body composition parameters and chronic LBP, several important knowledge gaps remain. Most investigations have evaluated these factors separately or have focused primarily on pain intensity, obesity, or general anthropometric measures. Relatively few studies have simultaneously evaluated body composition characteristics, MWR, pain intensity, and functional disability using multivariable analytical models within the same cohort of patients with chronic non-specific LBP while also exploring potential sex-related differences. Therefore, despite growing evidence linking skeletal muscle health and body composition to chronic non-specific LBP, the adjusted association between skeletal muscle status and functional disability after adjustment for pain intensity and relevant body composition variables remains incompletely understood. Furthermore, sex-related differences in these associations have received limited investigation.

We hypothesized that lower MWR values and higher pain intensity would remain associated with greater functional disability after adjustment for relevant covariates. We further hypothesized that female participants would exhibit higher pain intensity and greater disability compared with male participants.

## 2. Materials and Methods

### 2.1. Study Design and Participants

This cross-sectional observational study was conducted to investigate the associations between body composition parameters, MWR, pain intensity, and functional disability in patients with chronic non-specific LBP. Participants were consecutively recruited from patients presenting to the Department of Physical Medicine and Rehabilitation of the Clinical Emergency Hospital “Avram Iancu”, Oradea, Romania, between January and December 2025. The diagnosis of chronic non-specific LBP was confirmed by a consultant in Physical Medicine and Rehabilitation after exclusion of specific spinal pathology based on clinical history and physical examination, and additional imaging was reviewed when clinically indicated.

Participants were eligible for inclusion if they met all of the following criteria:

- Age ≥ 18 years;
- Clinical diagnosis of chronic non-specific LBP, defined as pain localized between the lower costal margin and the gluteal folds, with or without referred leg pain, persisting for more than 12 weeks;
- Absence of a specific spinal pathology explaining the symptoms (e.g., vertebral fracture, spinal infection, malignancy, inflammatory spinal disease, cauda equina syndrome, or other serious spinal pathology;
- Ability to stand independently for anthropometric and body composition measurements;
- Ability to understand and complete the study questionnaires;
- Provision of written informed consent.

Participants were excluded if they met any of the following criteria:

- Acute traumatic spinal injury;
- Vertebral fracture;
- Spinal infection;
- Inflammatory spinal disease;
- Active malignant disease;
- Cauda equina syndrome;
- Severe neurological disorders affecting mobility or balance;
- Presence of implanted electronic devices contraindicating bioelectrical impedance analysis (BIA) (e.g., pacemakers);
- Severe cognitive impairment preventing reliable questionnaire completion;
- Pregnancy;
- Incomplete clinical, anthropometric, or questionnaire data.

**Figure 1:**
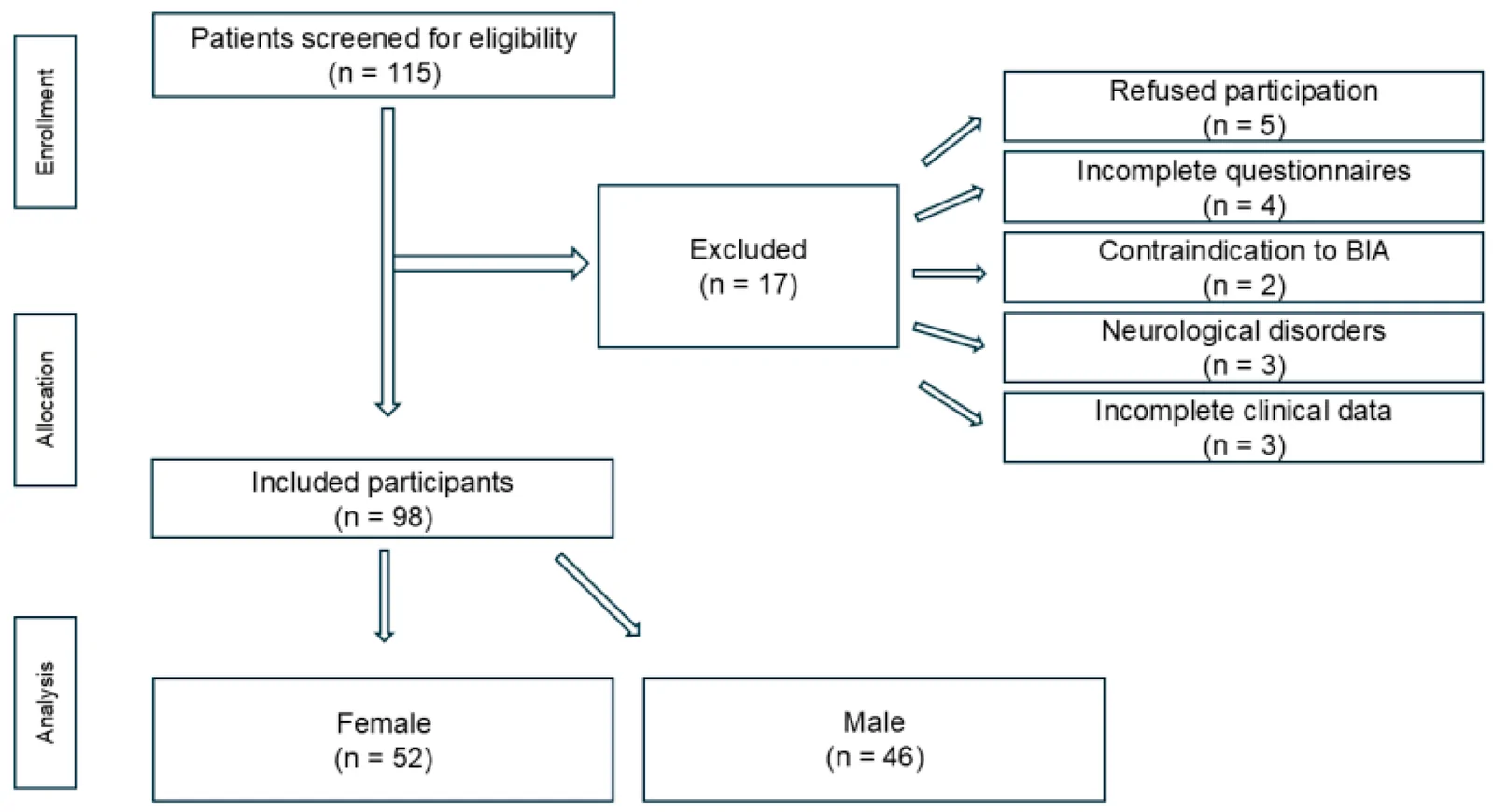
Flow diagram of participant selection and inclusion in the study. Abbreviations: BIA: Bioelectrical Impedance Analysis.

A total of 98 participants met the eligibility criteria and were included in the final analysis. Demographic characteristics, anthropometric measurements, body composition parameters, pain intensity scores, and disability assessments were collected during a single study visit. The participant selection process and final study population are presented in Figure 1.

### 2.2. Anthropometric and Body Composition Assessment

Anthropometric and body composition measurements were performed during a single study visit by trained healthcare personnel following standardized procedures.

Body weight (kg) and body composition parameters were assessed using a BIA device Tanita MC-580 MA (Tanita Corporation, Tokyo, Japan). Participants were evaluated barefoot and wearing light clothing, according to the manufacturer’s recommendations. Height (cm) was measured using a wall-mounted stadiometer, and BMI was subsequently calculated as weight in kilograms divided by height in meters squared (kg/m^2^).

The following body composition parameters were recorded:

- Body fat percentage (%);
- Muscle mass (kg);
- Bone mass (kg);
- Total body water percentage (%).

The MWR was calculated and used as an indicator of relative muscle status. Higher values were considered indicative of more favorable muscle composition.

To minimize measurement variability, all body composition assessments were performed by trained healthcare personnel following standardized procedures. Participants were instructed to avoid vigorous physical activity for at least 24 h before the assessment, refrain from alcohol consumption during the preceding 24 h, avoid eating or drinking for at least 2 h before the measurement, and empty their bladder immediately before testing. Whenever possible, measurements were performed in the morning. All assessments were conducted with participants barefoot and wearing light clothing according to the manufacturer’s recommendations.

### 2.3. Pain and Disability Assessment

Pain intensity was assessed using a Visual Analogue Scale (VAS). Participants were asked to rate their average LBP intensity on a 100 mm horizontal line ranging from 0 (“no pain”) to 100 (“worst imaginable pain”). Higher scores indicated greater pain intensity.

Functional disability was evaluated using the Roland–Morris Disability Questionnaire (RMDQ), a widely used and validated self-report instrument designed to assess disability associated with LBP. The questionnaire consists of 24 items related to physical functioning and daily activities. Participants were instructed to indicate the statements that best described their condition on the day of assessment.

RMDQ scores range from 0 to 24, with higher scores indicating greater disability. For descriptive purposes, RMDQ scores were categorized into five disability severity levels to facilitate interpretation of disability severity within the study population. These categories were defined by the authors solely for descriptive presentation and were not intended to represent validated clinical cut-off values. All inferential statistical analyses were performed using the continuous RMDQ score. For descriptive analyses, disability severity was categorized as follows:

Minimal or no disability: 0–3 points;

- Mild disability: 4–8 points;
- Moderate disability: 9–12 points;
- Severe disability: 13–16 points;
- Very severe disability: 17–24 points.

Pain intensity and disability assessments were completed during the same study visit as the anthropometric and body composition measurements.

### 2.4. MWR Calculation

The MWR was calculated as the ratio of skeletal muscle mass to total body weight and expressed as a percentage using the following formula:

(1) MWR = (Skeletal Muscle Mass (kg)/Body Weight (kg)) × 100

The MWR was used as an indicator of relative muscle status, reflecting the proportion of skeletal muscle mass in relation to total body weight. In the present study, MWR was defined as skeletal muscle mass divided by body weight × 100 and expressed as a percentage. This variable reflects weight-adjusted skeletal muscle mass and was used as an indicator of relative muscle status. The weight-adjusted MWR was selected because it reflects the proportion of skeletal muscle relative to total body weight and has been widely used in studies evaluating relative skeletal muscle mass and body composition in epidemiological and clinical populations. Compared with absolute muscle mass alone, this index better accounts for differences in body size and adiposity and provides skeletal muscle mass relative to body weight [29,30,31]. Higher MWR values indicate a more favorable muscle composition, whereas lower values suggest reduced relative muscle mass and a potentially increased risk of functional impairment. This index should not be interpreted as a diagnostic measure of sarcopenia.

The calculated MWR values were subsequently included in the descriptive, correlation, and multivariable regression analyses to evaluate their association with functional disability in patients with chronic non-specific LBP. Because no validated cut-off values were applied, participants were not classified as having or not having sarcopenia.

### 2.5. Statistical Analysis

Statistical analyses were performed using IBM SPSS Statistics software (Version 29.0; IBM Corp., Armonk, NY, USA). Continuous variables were assessed for normality using graphical methods and the Shapiro–Wilk test. Descriptive statistics are presented as mean ± standard deviation (SD) for continuous variables and as frequencies and percentages for categorical variables.

Comparisons between male and female participants were performed using the independent-samples *t*-test for normally distributed continuous variables. Associations between categorical variables were initially assessed using Pearson’s chi-square test. Expected cell frequencies were examined to verify the assumptions of the chi-square test. When more than 20% of cells had expected counts below 5, the Fisher–Freeman–Halton exact test was used. The strength of association between sex and disability severity was summarized using Cramer’s V.

Pearson correlation analysis was conducted to examine the relationships between RMDQ scores and demographic, anthropometric, body composition, and clinical variables, including age, BMI, body fat percentage, muscle mass, bone mass, total body water percentage, MWR, and pain intensity.

Before performing the multiple linear regression analysis, the assumptions of linear regression were evaluated. Multicollinearity among the independent variables was assessed using tolerance values and the Variance Inflation Factor (VIF). A tolerance value > 0.20 and a VIF < 5 were considered indicative of the absence of problematic multicollinearity. The assumptions of linearity, normality of residuals, homoscedasticity, and independence of residuals were also evaluated using residual plots and the Durbin–Watson statistic.

A multiple linear regression analysis was performed to evaluate the association between MWR and functional disability after adjustment for potential confounders. Age, sex, pain intensity, body fat percentage, and MWR were selected a priori based on clinical relevance, the study objectives, and previous literature. Age, sex, and pain intensity were included because of their established relationships with disability in chronic LBP. Body fat percentage was included as an indicator of adiposity, while MWR represented the primary muscle-related exposure. Body weight and absolute muscle mass were not entered because they are direct components of the MWR calculation. Although MWR and body fat percentage are mathematically related because both are derived from body composition measurements, some degree of mathematical coupling between these variables is unavoidable. Nevertheless, body fat percentage was retained in the model to adjust for adiposity while evaluating the association between MWR and disability. Potential multicollinearity was assessed using tolerance values and the VIF, and no evidence of problematic multicollinearity was observed. BMI was not included simultaneously with body fat percentage because both represent related measures of body size and adiposity. Total body water was excluded because it is closely related to lean tissue and hydration status, while bone mass was excluded because it was not considered a clinically plausible confounder of the relationship between MWR and disability. A sensitivity analysis was performed by replacing body fat percentage with BMI. These findings indicate that the observed association between MWR and disability was not explained solely by obesity, as similar results were obtained after adjustment using either body fat percentage or BMI. Because correlation analysis evaluates only unadjusted pairwise associations, variables were selected for multivariable regression based on clinical relevance and potential confounding rather than solely on statistical significance in univariate analyses.

An a priori sample-size calculation was performed using G*Power software (version 3.1.9.7; Heinrich Heine University Düsseldorf, Düsseldorf, Germany) for a multiple linear regression model with five predictors. Because no previous study reported an effect estimate directly applicable to our planned regression model, a conventional medium effect size (f^2^ = 0.15), as recommended by Cohen for multiple regression when prior effect estimates are unavailable, was assumed a priori. With a two-sided α level of 0.05 and 80% power, the minimum required sample size was 92 participants. Recruitment continued beyond this minimum to account for potential exclusions, and 98 participants with complete data were included in the final analysis [32,33].

All statistical tests were two-tailed, and a *p*-value < 0.05 was considered statistically significant.

### 2.6. Ethical Considerations

The study was conducted in accordance with the ethical principles of the Declaration of Helsinki and its subsequent amendments. Ethical approval was obtained from the Ethics Committee of the Clinical Emergency Hospital “Avram Iancu”, Oradea, Romania (Approval No. 1118263/30/05/2024).

Prior to participation, all eligible individuals received detailed information regarding the study objectives and procedures. Written informed consent was obtained from all participants before enrollment. Participation was voluntary, and all data were collected and analyzed anonymously to ensure participant confidentiality and privacy.

The study involved non-invasive assessments routinely used in clinical practice, including anthropometric measurements, BIA, and standardized self-report questionnaires. No experimental interventions were performed.

## 3. Results

### 3.1. Participant Characteristics

A total of 98 patients with chronic non-specific LBP were included in the study. All participants had symptoms persisting for more than 12 weeks and were referred for conservative rehabilitation management. None presented with clinical evidence of serious spinal pathology requiring surgical or emergency management. The mean age of the participants was 61.9 ± 10.0 years, and females represented 53.1% of the study population. The mean BMI was 33.5 ± 6.4 kg/m^2^. Mean body fat percentage, muscle mass, bone mass, and total body water were 35.7 ± 8.7%, 39.3 ± 12.7 kg, 2.67 ± 0.45 kg, and 37.8 ± 7.8%, respectively. The mean RMDQ score was 6.68 ± 5.86 points. Detailed demographic, anthropometric, body composition, and functional characteristics of the study population are presented in Table 1.

### 3.2. Sex-Related Differences

Comparisons of anthropometric and body composition parameters according to sex are presented in Table 2. Male participants were significantly taller (174.5 ± 6.6 cm vs. 159.3 ± 7.0 cm, *p* < 0.001) and heavier (97.4 ± 18.4 kg vs. 84.8 ± 16.7 kg, *p* < 0.001) than female participants. No significant differences were observed in age (*p* = 0.195) or BMI (*p* = 0.379).

Men demonstrated significantly greater muscle mass (41.7 ± 13.6 kg vs. 37.2 ± 11.5 kg, *p* = 0.045) and bone mass (4.19 ± 1.02 kg vs. 2.68 ± 0.60 kg, *p* < 0.001). Total body water percentage was also significantly higher among males (48.2 ± 7.6% vs. 38.1 ± 5.2%, *p* < 0.001). In contrast, females exhibited significantly higher body fat percentage compared with males (37.4 ± 7.9% vs. 33.8 ± 9.2%, *p* = 0.040).

**Figure 2:**
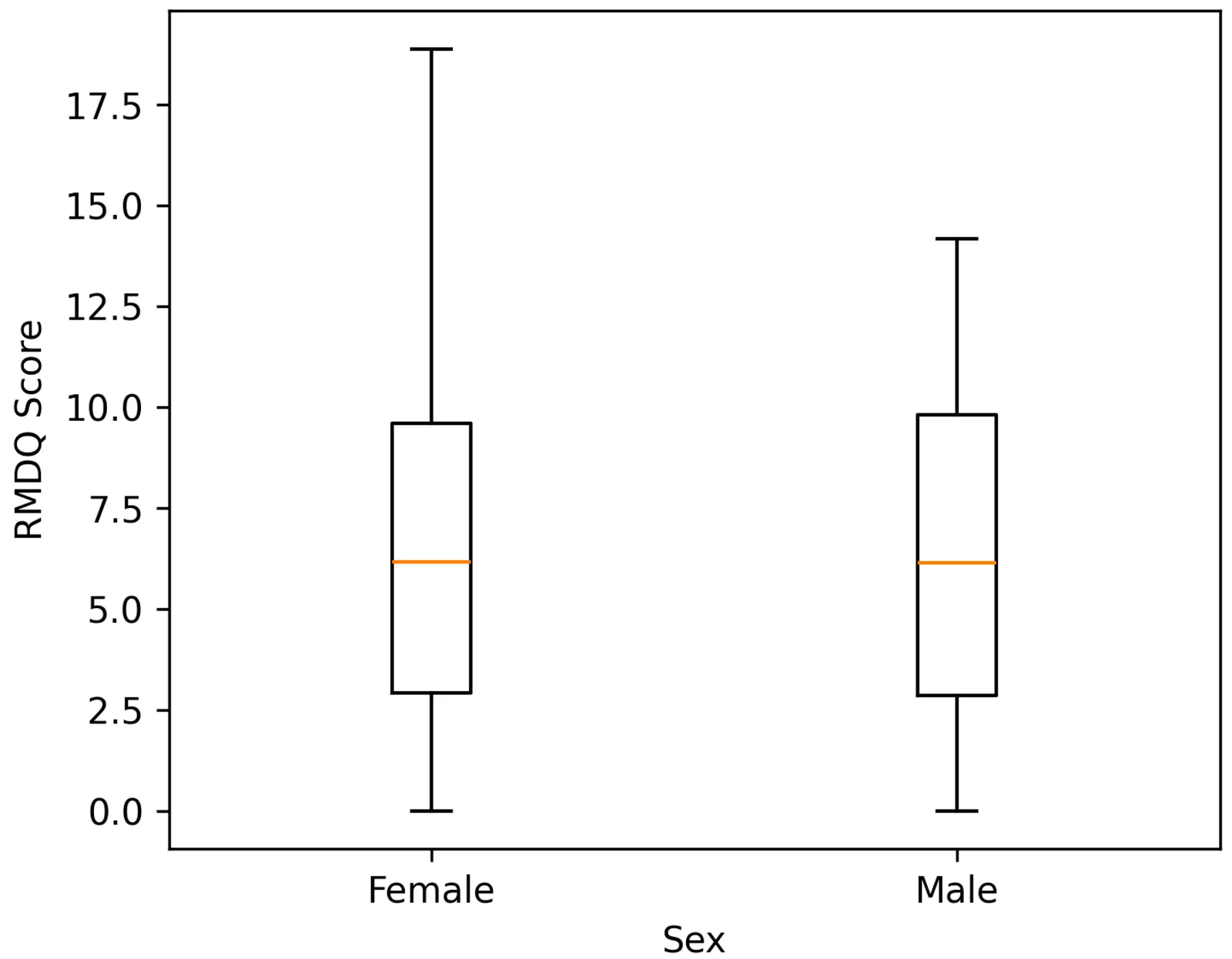
Distribution of RMDQ scores according to sex. Abbreviations: RMDQ: Roland–Morris Disability Questionnaire.

Female participants reported higher disability scores than male participants (7.59 ± 6.10 vs. 5.65 ± 5.45 points); however, this difference did not reach statistical significance (*p* = 0.101). The distribution of RMDQ scores according to sex is illustrated in Figure 2.

### 3.3. Functional Disability Distribution

The distribution of participants according to RMDQ severity categories is presented in Table 3. Overall, 41.8% of the patients were classified as having minimal or no disability (RMDQ score 0–3), whereas 22.5% demonstrated mild disability. Moderate disability was observed in 12.2% of the study population. Severe and very severe disability were identified in 16.3% and 7.2% of participants, respectively.

Overall, approximately one-quarter of the study population (23.5%) presented severe or very severe disability, indicating substantial functional impairment associated with LBP.

**Figure 3:**
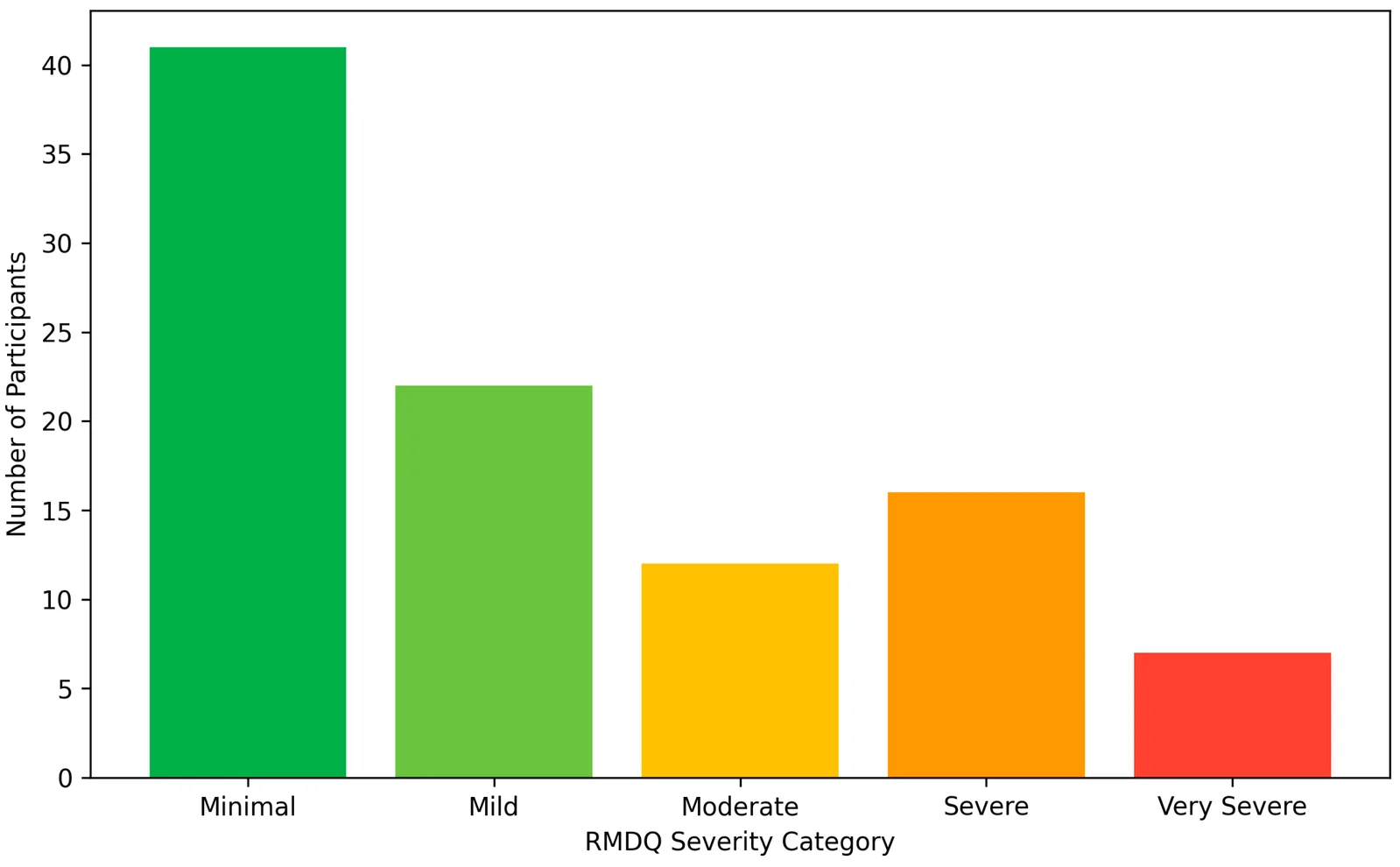
Distribution of disability severity categories according to RMDQ scores. Abbreviations: RMDQ: Roland–Morris Disability Questionnaire.

The distribution of disability severity categories within the study population is illustrated in Figure 3.

### 3.4. Association Between Disability Severity and Sex

The distribution of disability severity categories according to sex is presented in Table 4. Female participants demonstrated a higher prevalence of severe disability categories compared with males. Notably, all cases classified as very severe disability were observed among female participants, whereas no male participant was included in this category. Conversely, male participants were more frequently classified within the minimal disability category.

Because 3 of the 10 cells (30%) had expected counts below 5, the assumptions of Pearson’s chi-square test were not fully satisfied. Therefore, the association between sex and disability severity was evaluated using the Fisher–Freeman–Halton exact test. The distribution of disability severity differed significantly between female and male participants (exact *p* = 0.021). The magnitude of the association was moderate according to Cramer’s V (V = 0.338), indicating a meaningful relationship between sex and disability severity.

These findings suggest that female participants experienced greater functional impairment associated with chronic non-specific LBP than male participants.

### 3.5. Pain Intensity According to Sex

Pain intensity scores differed significantly between female and male participants. Female participants reported higher pain levels compared with males (60.54 ± 31.02 vs. 44.20 ± 35.52 points, *p* = 0.021).

These findings indicate that women experienced greater pain intensity than men within the study population. Descriptive statistics for pain scores according to sex are presented in Table 5.

### 3.6. Correlation Analysis

Correlation analyses were performed to investigate the relationships between disability, assessed using the RMDQ, and demographic, anthropometric, body composition, and clinical variables. The results are presented in Table 6.

Pain intensity demonstrated the strongest association with disability, showing a moderate positive correlation with RMDQ scores (r = 0.56, *p* < 0.001). This finding indicates that participants reporting higher pain levels also tended to experience greater functional impairment.

The MWR was negatively correlated with disability (r = −0.34, *p* = 0.001), suggesting that better muscle status was associated with lower disability scores. Weak but statistically significant correlations were also observed for body fat percentage (r = 0.21, *p* = 0.040) and total body water percentage (r = −0.22, *p* = 0.030). Higher body fat was associated with increased disability, whereas higher total body water was associated with lower disability levels.

No significant correlations were identified between RMDQ scores and age, BMI, muscle mass, or bone mass (all *p* > 0.05).

The observed correlations suggest that both pain intensity and muscle-related parameters may contribute to functional disability among patients with chronic non-specific LBP. Variables were considered for inclusion in the multivariable regression model based on clinical relevance, the study objectives, previous literature, and their potential role as confounders, rather than solely on the statistical significance of univariate associations.

### 3.7. Multiple Linear Regression Analysis

Prior to regression analysis, the assumptions of multiple linear regression were assessed. No evidence of problematic multicollinearity was observed among the predictor variables, with tolerance values ranging from 0.50 to 0.80 and VIF values ranging from 1.25 to 2.00 (Table 7). Collinearity diagnostics further demonstrated no evidence of harmful multicollinearity, with a maximum condition index of 15.1. Visual inspection of residual plots demonstrated no meaningful deviations from the assumptions of linearity, homoscedasticity, or normality of residuals. The Durbin–Watson statistic was 1.94, indicating no evidence of autocorrelation.

A multiple linear regression analysis was performed to evaluate the association between MWR and functional disability after adjustment for age, sex, body fat percentage, and pain intensity. The overall regression model was statistically significant (F = 13.8, *p* < 0.001) and explained 43.2% of the variance in disability scores (R^2^ = 0.432; adjusted R^2^ = 0.398).

Pain intensity remained the variable most strongly associated with disability (β = 0.49, *p* < 0.001). Age was positively associated with disability (β = 0.22, *p* = 0.013), whereas MWR remained inversely associated with disability after adjustment for the included covariates (β = −0.27, *p* = 0.018). Body fat percentage (β = 0.09, *p* = 0.240) and sex (β = 0.11, *p* = 0.180) were not significantly associated with disability in the adjusted model.

A sensitivity analysis was performed by replacing body fat percentage with BMI. The results were consistent with those of the primary model. MWR remained significantly and inversely associated with disability (β = −0.26, *p* = 0.020), whereas BMI was not significantly associated with disability (β = 0.08, *p* = 0.310). The sensitivity model remained statistically significant (R^2^ = 0.429; adjusted R^2^ = 0.395; F = 13.5; *p* < 0.001), indicating that the observed association between MWR and disability was robust regardless of whether adiposity was represented by body fat percentage or BMI.

Overall, these findings suggest that pain intensity, age, and MWR are important factors associated with functional disability among patients with chronic non-specific LBP. MWR remained significantly and inversely associated with disability after adjustment for the included covariates. Detailed regression coefficients are presented in Table 8.

Although age was not significantly correlated with disability in the univariate analysis, it became significantly associated with disability in the multivariable regression model after adjustment for sex, pain intensity, body fat percentage, and MWR. This finding suggests that the relationship between age and disability may have been influenced by confounding among the included variables. The regression model estimates the association between age and disability while holding the remaining covariates constant, which may reveal associations that are not evident in unadjusted pairwise correlation analyses.

## 4. Discussion

The present study investigated the relationships between body composition characteristics, MWR, pain intensity, and functional disability among patients with chronic non-specific LBP. Several important findings emerged. First, female participants demonstrated higher pain intensity and greater disability compared with male participants. Second, pain intensity was strongly associated with functional disability. Third, the MWR was negatively associated with disability, indicating that poorer muscle status was related to greater functional impairment. Finally, multivariable analysis showed that pain intensity, age, and MWR remained significantly associated with disability after adjustment for the included covariates. Pain intensity showed the strongest adjusted association with disability. Collectively, these findings suggest that functional disability in chronic non-specific LBP is associated not only with pain intensity but also with muscle-related factors. However, because of the cross-sectional design, these findings should not be interpreted as evidence of causal relationships.

### 4.1. Pain and Functional Disability

The present findings indicate that pain intensity was strongly associated with functional disability among individuals with chronic non-specific LBP. Pain intensity showed the strongest adjusted association with disability. These results are consistent with previous studies showing that greater pain severity is associated with physical limitations, activity restriction, and poorer quality of life in patients with chronic non-specific LBP [34,35,36].

Although the present study cannot establish causal mechanisms, previous studies have proposed several explanations for this observed association. In addition, pain-related fear and avoidance behaviors may lead patients to restrict daily activities, resulting in reduced physical activity, impaired trunk muscle activation, and progressive muscle deconditioning. Repeated avoidance of movement may also decrease confidence in performing functional tasks and reinforce disability-related behaviors. Over time, these interacting processes may create a self-perpetuating cycle in which pain reduces activity, reduced activity worsens physical capacity, and declining physical capacity further increases functional limitation [37,38].

The strong association observed in the present study therefore suggests that pain-related disability is likely influenced by factors beyond pain intensity alone, but also by the behavioral and physical consequences of persistent pain. These findings are consistent with current rehabilitation approaches that combine pain management with graded physical activity, restoration of movement confidence, and interventions targeting fear-avoidance and physical deconditioning.

The apparent discrepancy between the non-significant univariate correlation and the significant adjusted association observed for age illustrates the importance of multivariable analyses. Pearson correlation evaluates the crude association between two variables, whereas multiple linear regression estimates the association after adjustment for other clinically relevant covariates. Consequently, variables that are not significant in univariate analyses may become significant after confounding has been controlled. After adjustment for pain intensity, body composition, sex, and MWR, age was significantly associated with disability despite the absence of a significant univariate correlation, suggesting that the crude association was influenced by confounding among the included variables.

### 4.2. MWR and Disability

An important finding of the present study was the significant association between MWR and disability. Participants with lower MWR values tended to exhibit higher RMDQ scores, and this relationship remained significant after adjustment for potential confounders.

These findings are consistent with previous studies reporting associations between skeletal muscle health and physical function [21,39,40,41]. Although clinically diagnosed sarcopenia has been associated with impaired balance, reduced muscular endurance, and disability, the present study evaluated only relative skeletal muscle mass rather than sarcopenia [41,42,43,44]. Several mechanisms may explain the observed association between lower MWR and greater disability. Skeletal muscle plays a fundamental role in maintaining spinal stability, absorbing mechanical loads, and generating the muscular force required for posture, walking, and other functional activities. One possible explanation proposed in previous studies is that lower relative muscle mass may be associated with reduced trunk stabilization, lower muscular endurance, and greater fatigue during daily activities. However, these potential mechanisms cannot be confirmed in the present cross-sectional study.

In addition, reduced skeletal muscle mass is frequently accompanied by decreased muscle strength, impaired neuromuscular control, and lower physical performance, all of which may contribute to difficulty performing routine activities and increased disability. It is possible that persistent pain is associated with lower levels of physical activity, which may in turn be accompanied by reduced muscle mass. Conversely, lower muscle mass may also be associated with greater disability. Because measurements were obtained at a single time point, the direction of these relationships cannot be determined.

In patients with chronic non-specific LBP, previous studies have suggested that deterioration of muscle quality may be associated with reduced spinal stability and movement efficiency [45,46,47,48,49].

Interestingly, conventional anthropometric indicators such as BMI and body fat percentage demonstrated weaker associations with disability than the MWR. This observation suggests that muscle-related parameters may provide clinically relevant information beyond body weight alone. Unlike BMI, which cannot distinguish between fat and lean tissue, the MWR provides information about the relative amount of skeletal muscle available to support movement and functional performance. Consequently, the MWR may provide additional information beyond BMI regarding muscle status and functional impairment than traditional measures of obesity alone. Similar findings have been reported in studies indicating that body composition quality is a stronger determinant of functional outcomes than traditional obesity measures [19,23,39,40,41]. An important consideration when interpreting the present findings is that the study population was predominantly obese, with a mean BMI of 33.5 kg/m^2^. Because MWR incorporates body weight in its calculation, lower values could theoretically reflect greater adiposity rather than reduced relative muscle mass alone. To address this possibility, multivariable models were adjusted for body fat percentage, and a sensitivity analysis replacing body fat percentage with BMI yielded comparable results. In both models, the association between MWR and disability remained statistically significant, suggesting that the observed relationship was not explained solely by obesity. Nevertheless, because the study population was predominantly obese, residual confounding related to obesity cannot be completely excluded, and these findings should be confirmed in more heterogeneous populations including normal-weight individuals.

### 4.3. Interpretation of Sex-Related Differences

Female participants reported significantly higher pain intensity and were more frequently classified within severe disability categories than males. Moreover, all participants categorized as having very severe disability were women.

These findings are consistent with previous studies reporting greater pain sensitivity, higher prevalence of chronic musculoskeletal pain, and increased disability among women [12,50,51,52,53]. Several biological and psychosocial mechanisms may contribute to these sex-related differences. Hormonal influences, particularly fluctuations in estrogen levels, have been associated with altered pain perception and modulation, potentially increasing susceptibility to persistent pain. Furthermore, women generally have lower absolute skeletal muscle mass than men, which may reduce muscular reserve and spinal support, particularly in the presence of chronic pain and age-related muscle loss.

Psychosocial factors may also influence pain perception and disability. Differences in pain coping strategies, fear-avoidance behaviors, psychological distress, and healthcare-seeking behavior have all been proposed as contributors to the greater burden of chronic musculoskeletal pain observed among women. These factors may interact with biological mechanisms, resulting in higher pain intensity, reduced physical activity, and greater functional impairment over time [52,54,55,56].

The observed findings highlight the importance of considering sex-specific factors when evaluating patients with chronic non-specific LBP and developing individualized treatment approaches. Recognizing these differences may help clinicians identify patients at greater risk of disability and support the development of personalized rehabilitation strategies that address both physical and psychosocial contributors to chronic pain.

### 4.4. Clinical Implications

The findings of this study have several important clinical implications for the assessment and management of patients with chronic non-specific LBP. First, the strong association between pain intensity and disability reinforces the importance of routine pain evaluation in clinical practice. Pain remains one of the most relevant determinants of functional impairment, and early identification of patients experiencing high pain levels may facilitate timely intervention and prevent further deterioration in physical function.

Second, the present findings suggest that assessment of body composition and relative skeletal muscle mass may provide clinically meaningful information beyond conventional anthropometric measures such as BMI. While BMI remains widely used in routine practice, it does not distinguish between fat mass and lean tissue and may therefore fail to identify patients with unfavorable muscle characteristics despite having similar body weight profiles. The observed association between lower MWR values and greater disability indicates that muscle-related parameters may represent important markers of functional vulnerability among individuals with LBP.

From a mechanistic perspective, improving skeletal muscle health may influence functional outcomes through several pathways. Greater muscle mass and strength contribute to improved spinal stability, enhanced movement efficiency, and increased tolerance to physical activity, thereby reducing mechanical stress on the lumbar spine during daily activities. In addition, maintaining adequate muscle mass may help interrupt the cycle of pain, inactivity, and progressive deconditioning that characterizes many patients with chronic non-specific LBP. Consequently, interventions targeting muscle preservation and rehabilitation may have benefits beyond improving body composition alone, potentially leading to better functional recovery and long-term disability prevention.

These findings support the integration of body composition assessment into the comprehensive evaluation of patients with chronic musculoskeletal conditions. Identifying individuals with reduced muscle status may allow clinicians to implement targeted interventions aimed at preserving or improving muscle health before substantial functional decline occurs. Such interventions may include progressive resistance exercise programs, individualized physiotherapy, nutritional counseling, optimization of protein intake, and multidisciplinary rehabilitation approaches designed to improve both physical function and quality of life.

Furthermore, the observed sex-related differences highlight the need for individualized management strategies. Female participants demonstrated higher pain intensity and a greater prevalence of severe disability categories, suggesting that women may represent a subgroup at increased risk of adverse functional outcomes. Greater attention to sex-specific factors may improve risk stratification and facilitate the development of more personalized treatment plans.

From a broader perspective, the findings emphasize the importance of moving beyond symptom-oriented management and adopting a more comprehensive approach that incorporates pain assessment, body composition evaluation, and functional status monitoring. Such an approach may improve the identification of high-risk patients and contribute to more effective prevention and rehabilitation strategies aimed at reducing disability associated with LBP.

Finally, given the aging of the population and the increasing prevalence of both LBP and age-related loss of skeletal muscle mass, the incorporation of muscle health assessment into routine clinical practice may represent an important step toward more personalized and preventive musculoskeletal care. Early recognition of unfavorable muscle composition profiles may help guide interventions aimed at reducing disability, preserve independence, and improve long-term functional outcomes.

### 4.5. Limitations

Several limitations should be considered when interpreting the findings of the present study. First, the cross-sectional design precludes the establishment of causal relationships or determination of the temporal sequence between body composition characteristics, relative skeletal muscle mass, pain intensity, and functional disability. Therefore, the observed associations should not be interpreted as evidence that changes in muscle status cause disability or vice versa. It remains unclear whether unfavorable body composition is associated with subsequent disability or whether disability-related reductions in physical activity are accompanied by changes in muscle status and body composition. Longitudinal and interventional studies are required to clarify these temporal and causal relationships.

Second, the study was conducted in a relatively modest sample of patients recruited from a single clinical setting. Although the sample size was sufficient to identify significant associations, the findings may not be fully generalizable to other populations, healthcare systems, or geographic regions. Furthermore, the study population was predominantly obese, reflecting the characteristics of patients referred to our rehabilitation clinic. Therefore, the findings may not be directly generalizable to normal-weight individuals with chronic non-specific LBP. Future multicenter studies involving larger and more diverse populations, including individuals across a broader range of BMI categories, are needed to confirm the external validity of the present findings.

Third, body composition was assessed using BIA. Although BIA is a practical, non-invasive, and widely used method in clinical settings, its accuracy may be influenced by hydration status, recent food intake, physical activity, and other physiological factors. More precise methods such as dual-energy X-ray absorptiometry (DXA), computed tomography (CT), or magnetic resonance imaging (MRI) could provide a more detailed evaluation of muscle and adipose tissue characteristics.

Another important limitation relates to the assessment of relative muscle mass. Although the present study evaluated the MWR as an indicator of relative muscle mass, sarcopenia itself was not directly assessed. According to current international consensus definitions, the diagnosis of sarcopenia requires the combined evaluation of muscle quantity, muscle strength, and physical performance. Because measures of muscle strength (e.g., handgrip strength) and physical performance (e.g., gait speed or chair stand tests) were not available, it was not possible to determine the presence or severity of sarcopenia in the study population. Therefore, the findings should be interpreted as describing associations between MWR and functional disability rather than associations with clinically diagnosed sarcopenia. Therefore, no conclusions regarding the presence or prevalence of sarcopenia can be drawn from the present study.

Furthermore, although all participants had chronic non-specific LBP, no subgroup analyses were performed according to the duration of chronic symptoms (e.g., 3–6 months versus >12 months), recurrent versus persistent pain, or the presence of radicular symptoms. These clinical characteristics may influence pain intensity, body composition, and functional disability and therefore could have affected the observed associations. In addition, several potentially important confounding factors were not evaluated, including physical activity levels, occupational demands, nutritional status, smoking habits, psychological factors (including depression, anxiety, fear-avoidance beliefs, and pain catastrophizing), pain medication use, and history of previous spine surgery. The absence of these variables may have resulted in residual confounding and should be considered when interpreting the findings.

Furthermore, because MWR is mathematically derived from body weight and body fat percentage represents a related aspect of body composition, some degree of mathematical coupling between these variables cannot be excluded. Although multicollinearity diagnostics indicated no evidence of problematic collinearity (all VIF values < 5), the observed independent association between MWR and functional disability should be interpreted with appropriate caution.

Additionally, the study relied on self-reported measures of pain intensity and disability. Although the RMDQ and pain scales are well-validated instruments, self-reported data remain susceptible to reporting bias, recall bias, and individual differences in symptom perception.

Finally, the observational nature of the study does not allow conclusions regarding the effectiveness of interventions aimed at improving muscle status or reducing disability. Therefore, whether modification of relative skeletal muscle mass can directly improve functional outcomes in patients with chronic non-specific LBP remains to be established through prospective interventional research.

Despite these limitations, the study provides additional evidence regarding the associations between pain intensity, body composition, relative skeletal muscle mass, and disability in patients with chronic non-specific LBP and highlights the potential importance of incorporating muscle-related assessments into routine clinical evaluation.

### 4.6. Future Directions

Future research should focus on clarifying the causal relationships between relative skeletal muscle mass, pain intensity, and functional disability in patients with chronic non-specific LBP. Because the present study employed a cross-sectional design, it remains unclear whether unfavorable muscle status contributes to the development of disability or whether disability-related reductions in physical activity subsequently led to reductions in relative skeletal muscle mass. Longitudinal cohort studies are therefore needed to investigate the temporal sequence of these associations and to identify potential pathways linking muscle health to functional outcomes.

Larger multicenter investigations involving more diverse populations would improve the external validity and generalizability of the findings. Such studies could also facilitate subgroup analyses according to age, sex, obesity status, pain duration, and clinical severity, thereby providing a more comprehensive understanding of factors influencing disability among patients with chronic non-specific LBP.

Future studies should further explore the role of muscle health using more comprehensive assessments of sarcopenia. In addition to body composition measures, the inclusion of objective indicators of muscle strength and physical performance, such as handgrip strength, gait speed, chair stand tests, and balance assessments, may provide a more complete evaluation of the relationship between sarcopenia and disability. The use of advanced imaging techniques, including DXA, CT, and MRI, may also help clarify the contribution of muscle quality and composition to functional impairment.

Interventional research represents another important area for future investigation. Randomized controlled trials should evaluate whether interventions targeting muscle health can improve functional outcomes in patients with chronic non-specific LBP. Potential approaches include resistance exercise programs, structured physical rehabilitation, nutritional optimization, protein supplementation, and multimodal interventions combining exercise, nutrition, and pain management strategies. Determining whether improvements in relative skeletal muscle mass translate into reductions in disability would have important implications for clinical practice.

Given the observed sex-related differences in pain intensity and disability, future studies should also investigate the biological, hormonal, psychological, and social factors underlying these disparities. A better understanding of sex-specific mechanisms may contribute to the development of personalized treatment strategies and improve outcomes for both male and female patients.

Furthermore, future research should incorporate additional variables that may influence disability, including physical activity levels, psychological factors, comorbidities, sleep quality, nutritional status, and inflammatory biomarkers. Integrating these factors into multidimensional models may provide a more comprehensive understanding of disability in patients with chronic non-specific LBP and facilitate the identification of individuals at greatest risk of poor functional outcomes.

Ultimately, future studies should aim to develop predictive models that combine clinical, functional, and body composition parameters to improve risk stratification and support personalized rehabilitation strategies for patients with chronic non-specific LBP.

## 5. Conclusions

In conclusion, the present study demonstrated that pain intensity, age, and MWR were significantly associated with functional disability among patients with chronic non-specific LBP. Pain intensity showed the strongest adjusted association for disability, while lower MWR values were independently associated with greater functional impairment, highlighting the potential importance of muscle health in this population. Because of the cross-sectional design, these findings should not be interpreted as evidence of causal relationships. Longitudinal studies are needed to determine whether changes in muscle status precede changes in disability.

Female participants reported higher pain intensity and exhibited a greater prevalence of severe disability categories compared with males, suggesting that sex-related factors may influence clinical outcomes in patients with chronic non-specific LBP.

The findings further indicate that body composition assessment may provide clinically relevant information beyond conventional anthropometric measures such as BMI. In particular, the MWR may represent a useful marker associated with disability.

Taken together, these results support the integration of pain assessment, body composition evaluation, and MWR screening into the routine clinical management of patients with chronic non-specific LBP. Such an approach may facilitate earlier identification of high-risk individuals and contribute to the development of more personalized rehabilitation strategies aimed at reducing disability and improving functional outcomes.

## Figures and Tables

**Table 1:** Baseline characteristics of the study population (n = 98).

Variable	Value
**Sex, *n* (%)**	
Female	52 (53.1)
Male	46 (46.9)
**Continuous variables, mean ± SD (range)**	
Age (years)	61.90 ± 10.01 (34–80)
Height (cm)	166.45 ± 10.21 (144–185)
Weight (kg)	90.68 ± 18.49 (46.3–145.9)
BMI (kg/m^2^)	33.51 ± 6.41 (19.1–50.5)
Body fat (%)	35.72 ± 8.69 (8.0–60.1)
Muscle mass (kg)	39.30 ± 12.67 (19.4–60.3)
Bone mass (kg)	2.67 ± 0.45 (1.7–4.2)
Total body water (%)	37.77 ± 7.80 (25.6–66.4)
MWR	43.3 ± 8.0 (20–65)
Pain score	52.9 ± 34.0 (0–100)
RMDQ score	6.68 ± 5.86 (0–20)

Abbreviations: BMI: Body Mass Index; RMDQ: Roland–Morris Disability Questionnaire; SD: Standard Deviation; MWR: Muscle-to-Weight Ratio.

**Table 2:** Comparison of anthropometric and body composition parameters according to sex.

Variable	Female (*n* = 52)	Male (*n* = 46)	*p*-Value
Age (years)	63.14 ± 10.05	60.50 ± 9.89	0.195
Height (cm)	159.31 ± 7.01	174.52 ± 6.61	<0.001
Weight (kg)	84.79 ± 16.67	97.36 ± 18.35	<0.001
BMI (kg/m^2^)	34.05 ± 6.23	32.90 ± 6.62	0.379
Body fat (%)	37.42 ± 7.93	33.82 ± 9.20	0.040
Muscle mass (kg)	37.16 ± 11.46	41.73 ± 13.63	0.045
Bone mass (kg)	2.68 ± 0.60	4.19 ± 1.02	<0.001
Total body water (%)	38.06 ± 5.18	48.23 ± 7.60	<0.001
MWR	43.8 ± 7.0	42.9 ± 8.5	0.67
Pain score	60.54 ± 31.02	44.20 ± 35.52	0.021
RMDQ score	7.59 ± 6.10	5.65 ± 5.45	0.101

Abbreviations: BMI: Body Mass Index; RMDQ: Roland–Morris Disability Questionnaire; MWR: Muscle-to-Weight Ratio.

**Table 3:** Distribution of participants according to disability severity categories.

RMDQ Category	Score Range	*n*	%
Minimal/No disability	0–3	41	41.8
Mild disability	4–8	22	22.5
Moderate disability	9–12	12	12.2
Severe disability	13–16	16	16.3
Very severe disability	17–24	7	7.2

Abbreviations: RMDQ: Roland–Morris Disability Questionnaire.

**Table 4:** Distribution of disability severity categories according to sex.

Disability Category	Female *n* (%)	Male *n* (%)
Minimal	18 (34.6)	25 (54.3)
Mild	11 (21.2)	13 (28.3)
Moderate	8 (15.4)	2 (4.3)
Severe	9 (17.3)	6 (13.0)
Very severe	6 (11.5)	0 (0.0)
Total	52 (100)	46 (100)

Note: Because 3 of the 10 cells (30%) had expected counts below 5, the assumptions of Pearson’s chi-square test were not fully satisfied. Therefore, the association between sex and disability severity was evaluated using the Fisher–Freeman–Halton exact test. The association was statistically significant (exact *p* = 0.021). The effect size was moderate (Cramer’s V = 0.338).

**Table 5:** Comparison of pain intensity according to sex.

Variable	Female (*n* = 52)	Male (*n* = 46)
Pain score	60.54 ± 31.02	44.20 ± 35.52

**Table 6:** Correlations between study variables and RMDQ score.

Variable	Correlation Coefficient (r)	*p*-Value
Age	0.12	0.24
BMI	0.18	0.08
Body fat (%)	0.21	0.04
Muscle mass (kg)	−0.15	0.14
Bone mass (kg)	−0.10	0.31
Total body water (%)	−0.22	0.03
MWR	−0.34	0.001
Pain score	0.56	<0.001

Abbreviations: BMI: Body Mass Index; MWR: Muscle-to-Weight Ratio.

**Table 7:** Collinearity diagnostics for the multiple linear regression model.

Predictor	Tolerance	VIF
Age	0.80	1.25
Sex	0.74	1.35
Body fat (%)	0.56	1.79
Pain score	0.78	1.28
MWR	0.50	2.00

Note: Maximum Condition Index = 15.1, indicating no evidence of harmful multicollinearity. Abbreviations: MWR: Muscle-to-Weight Ratio; VIF: Variance Inflation Factor.

**Table 8:** Multiple linear regression model predicting RMDQ score.

Predictor	β	*p*
Age	0.22	0.013
Sex	0.11	0.180
Body fat (%)	0.09	0.240
Pain score	0.49	<0.001
MWR	−0.27	0.018

Note: Primary model statistics: R^2^ = 0.432; adjusted R^2^ = 0.398; F = 13.8; *p* < 0.001. Abbreviations: MWR: Muscle-to-Weight Ratio.

## Data Availability

The data presented in this study are available from the corresponding author upon reasonable request. The data are not publicly available due to privacy and ethical restrictions related to participant confidentiality.

## Abbreviations

The following abbreviations are used in this manuscript:

BIA	Bioelectrical Impedance Analysis
BMI	Body Mass Index
LBP	Low Back Pain
RMDQ	Roland–Morris Disability Questionnaire
SD	Standard Deviation
MWR	Muscle-to-Weight Ratio
VAS	Visual Analogue Scale
CT	Computed Tomography
DXA	Dual-Energy X-ray Absorptiometry
MRI	Magnetic Resonance Imaging
VIF	Variance Inflation Factor
